# An empirical study on ‘how to retain rural teachers with emphasis on hygiene or motivation factors’: A case of Western China

**DOI:** 10.3389/fpsyg.2023.1114107

**Published:** 2023-02-06

**Authors:** Jin-Qiu Jiang, Jie Yao, Kai-Rui Yu, Chong-Nan Li

**Affiliations:** School of Urban Economics and Public Administration, Capital University of Economics and Business, Beijing, China

**Keywords:** motivation factors, hygiene factors, turnover intention, rural teachers, teacher retention

## Abstract

How to retain prominent teachers is a fundamental problem for rural education in less developed countries. However, the critical institutional factors affecting teachers’ turnover intention still need to be better understood. According to Herzberg’s motivation-hygiene theory, this study examines the effects of hygiene factors (rural incentive policy, personnel policy, and teacher pay) and motivation factors (advancement, work itself, and achievement) on rural teachers’ turnover intention. Based on a sample of 973 rural teachers, the results from structural equation modeling (SEM) showed that both hygiene factors and motivation factors can significantly reduce the turnover intention of rural teachers. Meanwhile, the effect of motivation factors is greater than that of hygiene factors. It was also confirmed that marital status, age, and teaching subject have a significant moderation effect on the relationship between motivation/hygiene factor and turnover intention, whereas gender has no significant moderation effect. Theoretical and practical implications for attracting and retaining rural teachers are discussed.

## Introduction

The construction of rural teachers is the foundation of the development of rural education, and rural teachers play a vital role in promoting the development of education in backward and poor areas. The “Report on the Development of China’s Rural Education (2019)” and the “Rural Teacher Support Plan (2015–2020)” show that the rural teaching staff is developing well, but the loss of outstanding in-service rural teachers has made it difficult to build a teaching team in some rural areas. The recruitment and retention of teachers in impoverished areas is still a worldwide problem.

To reduce the tendency of rural teachers to leave, the Chinese government has formulated and promulgated various forms of teacher employment policies. For example, the 2015 “Rural Teacher Support Plan (2015–2020)” calls for strengthening support for rural teachers in remote and impoverished areas. In 2018, the “Implementation Plan for Educational Poverty Alleviation in Deeply Poverty Areas (2018–2020)” further emphasized that it is necessary to strengthen the construction of rural teachers through the in-depth implementation of the rural teacher support plan. Although China has adopted a large number of rural incentive strategies, what is the effect of rural teachers’ incentive policies? Do rural teacher support policies have a positive impact on teacher retention? What kind of incentive strategies can effectively reduce teachers’ turnover intention? Based on the above thinking, the research team conducted a questionnaire survey and in-depth interviews with in-service teachers working in rural schools in western China in May 2018.

The difference between this study and the previous literature is that the research objects are precisely positioned as in-service teachers working in rural schools in western China. The selection of teachers in schools in western China is based on practical considerations because the locations of schools in rural western China are remote. As well as the slow economic development, the problem of the loss of in-service teachers in rural areas is the most representative. Second, according to Herzberg’s motivation-health theory, institutional factors are divided into two categories: hygiene factors and motivation factors, in which hygiene factors represent lower-level needs, such as physiological and safety needs; motivation factors represent higher-level needs, such as social needs, esteem, and self-actualization needs, to verify and compare the differential effects of hygiene factors and motivation factors on rural teachers’ turnover intention (Sanjeev and Surya, 2016). The third is that rural teachers with different personal characteristics will prefer different incentive methods. This paper will further test the moderating role of teachers’ characteristics between motivation-hygiene factors and turnover intention, which will provide a reference for the recruitment and retention of rural teachers in western China.

## Theories and hypotheses

### Motivation-hygiene theory

The motivation-hygiene theory divides work motivation into two factors: motivation and hygiene (Herzberg et al., 1959). Among them, hygiene factors are related to a person’s basic survival needs, such as the reward system, wages, and interpersonal relationships. However, these factors cannot motivate or bring satisfaction to employees, they can only prevent dissatisfaction and maintain the original work efficiency and psychological state of employees. Motivation factors involve growth needs, which refer to internal factors such as the work itself, such as recognition of work completion, sense of accomplishment, responsibility, progress, and the work itself. These factors represent higher-level needs and are an important way to improve job satisfaction and thus enhance job motivation (Herzberg, 1971; Tietjen and Myers, 1998). If motivation factors can be satisfied, employees will be satisfied and improve their work enthusiasm and efficiency. If motivation factors are not met, they will not create negative emotions in employees like hygiene factors. For example, a person who leaves a teaching job they love because they do not earn enough to cover living expenses (hygiene factor), or a person who leaves a teaching job that pays well but they do not like teaching every day (motivation factor). Looking at this example, the ideal situation would be to hire someone who intrinsically enjoys teaching and is paid well enough not to worry about the salary. “The modern strategic human resources management (HRM) practices, argue that school leaders should intentionally design a supportive employee experience for teacher support as an organizational talent management strategy” (Tran and Smith, 2020). A major cause of teacher turnover is insufficient employer support for teachers’ needs, i.e., administrative support has been identified as the most important factor for teacher retention (Ingersoll, 2001; Horng, 2009), so we focus on administrative support strategies.

### Research hypothesis

What factors affect the turnover intention of rural teachers? The motivation-hygiene theory states that teachers, when choosing a teaching career, are influenced by hygiene factors related to needs at lower levels in the hierarchy of needs - i.e. money, working conditions, relationships or company policies, etc., as well as motivation factors related to needs at higher levels - promotion, achievement, responsibility, etc. On the whole, rural teachers’ turnover intention is affected by institutional factors including teachers’ salaries, working conditions, policy formulation and implementation, and individual characteristics including gender, age, experience, and ability (Hanushek and Rivkin, 2010; Ping and Yao, 2019).

### Hygiene factors and turnover intention

According to Herzberg’s motivation-hygiene theory, hygiene factors represent lower levels of needs, and when these factors are lower than the teacher’s acceptable level, teachers will become dissatisfied with their jobs and even leave their jobs (Sanjeev and Surya, 2016). Based on the survey of rural teachers in western China, this paper focuses on the analysis of three important hygiene factors that rural teachers are most concerned about, namely rural incentive policies, personnel policies, and teacher pay.

The reason for choosing these three hygiene factors is that the Chinese government and schools have reformed these three aspects of hygiene factors for rural teachers, and we pay attention to the impact of these policy interventions.

#### Rural incentive policies

The rural incentive policy is chosen because China has introduced a series of incentive policies for rural teachers since 2013 to attract and retain rural teachers, such as the “Living Subsidy Policy for Teachers in Poor Areas (2013),” “Rural Teacher Support Program (2015–2020),” “Solid Implementation Plan for Education in Deeply Impoverished Areas (2018–2020).” which emphasizes that provincial and county governments should design monetary and non-monetary incentive policies for rural teachers according to their conditions. However, research shows that since there is a financial gap between different regions in China, the subsidy standard between counties and provinces is between 100 and 3,000 yuan per month. As a result, rural teachers respond to incentives that differ from county to county, making appropriate decisions to stay or leave. However, few empirical studies focus on the effect of these incentive policies. If these policies are effective, they can provide a basis for the improvement of future policies and inspire other developing countries.

Teacher-related policy development and implementation can affect teachers’ turnover intentions. For example, incentives such as bonuses, housing subsidies, and loan forgiveness can reduce teachers’ decisions to leave (Loeb et al., 2009; Anzia and Moe, 2013).

#### Personnel policy

Personnel policies also affect rural teachers’ decisions to leave. The existing research lacks a unified theoretical framework, which makes the formulation of related policies more scattered and lacks a systematic plan. Teachers are more willing to work in schools with reasonable evaluation systems, fair performance-based pay programs, and more opportunities for promotion. In addition, if a county has a flexible policy of swapping jobs between rural and urban teachers, rural teachers are more likely to stay in their current positions and continue to work.

Since the implementation of the merit pay reform in 2019 in China, there have been differences in the performance-based salary evaluation and distribution of primary and secondary schools. Each school has its performance distribution plan which is related to teacher salary distribution and promotion. It will have an impact on their turnover intentions. Therefore, personnel policy is also one of the key hygiene factors affecting rural teachers’ decisions to leave.

#### Teacher pay

Teacher pay has a key impact on rural teachers’ career choices, professional identity, and career development. Research shows that higher wages will reduce rural teachers’ turnover intention. The higher the salary, the longer the teachers are willing to stay (Imazeki, 2005). Since the 1980s, rural teacher pay has been funded by township governments, and rural teachers in impoverished areas cannot even get their monthly wages on time. This problem was not solved until the education reform in 2001 when the responsibility for providing basic education was transferred to the county government. However, there are still large county-province differences in rural teacher pay in China (Jiang and Yuhong, 2012). In addition, although rural teachers receive a certain monthly living allowance, there are the following problems when issuing living allowances: the effect of attracting outstanding teachers to teach in remote rural areas is limited; the county-level financial burden is increased in most underdeveloped areas; rural and remote areas in underdeveloped areas being easily overlooked in the process of granting subsidies (Jingxun and Renfang, 2018). Therefore, teacher pay is still a hygiene factor that rural teachers value in China, and raising teacher pay can reduce teachers’ willingness to leave (Shanhuai et al., 2018; Opoku et al., 2019).

Therefore, this study proposes the following hypotheses:

*H1*: Hygiene factors composed of rural incentive policies, personnel policies, and teachers' wages are negatively correlated with rural teachers' turnover intention.

### Motivation factors and turnover intention

Motivation factors can meet the personal psychological and growth needs of employees and are an important way to improve employee job satisfaction and reduce turnover intentions. Among them, promotion, work itself, and achievement are the key motivating factors (Sanjeev and Surya, 2016; Hur, 2018).

#### Promotion

Training promotes employee growth and provides learning opportunities and professional development to help retain employees (McGilton et al., 2013). Therefore, rural schools can improve teachers’ teaching skills by providing in-school training courses such as teacher training and mentoring, which is conducive to reducing the turnover rate of teachers.

#### The work itself

The job itself generally refers to an employee’s relationship with a customer or group of customers inside or outside the organization. If rural teachers can establish good relationships with students, students’ parents, and rural communities, they will have the motivation to stay with students and strive to adapt to the rural school environment and living conditions (Boyd et al., 2011; Goodpaster et al., 2012).

#### Achievements

When employees gain self-achievement and recognition from the perspective of work, professional satisfaction will appear and be further transformed into intrinsic motivation, which can promote teachers’ mental health, enhance teachers’ participation, and prompt teachers to choose to continue their current work and continue Retaining teachers will demonstrate higher teaching effectiveness (Kang et al., 2015; Abós et al., 2018; Casely-Hayford et al., 2022). Recognizing employees’ efforts and contributions is an effective and low-cost method to keep employees’ commitment to the organization, so rural teachers in schools that give respect, recognition, and appreciation to teachers have relatively low turnover rates (Ashiedu and Scott-Ladd, 2012; Hogan et al., 2013).

The above discussion leads to the second hypothesis:

*H2*: Motivation factors consisting of promotion, work itself, and achievement were significantly negatively correlated with rural teachers' turnover intention.

### The moderating effect of individual characteristics

**Figure 1:**
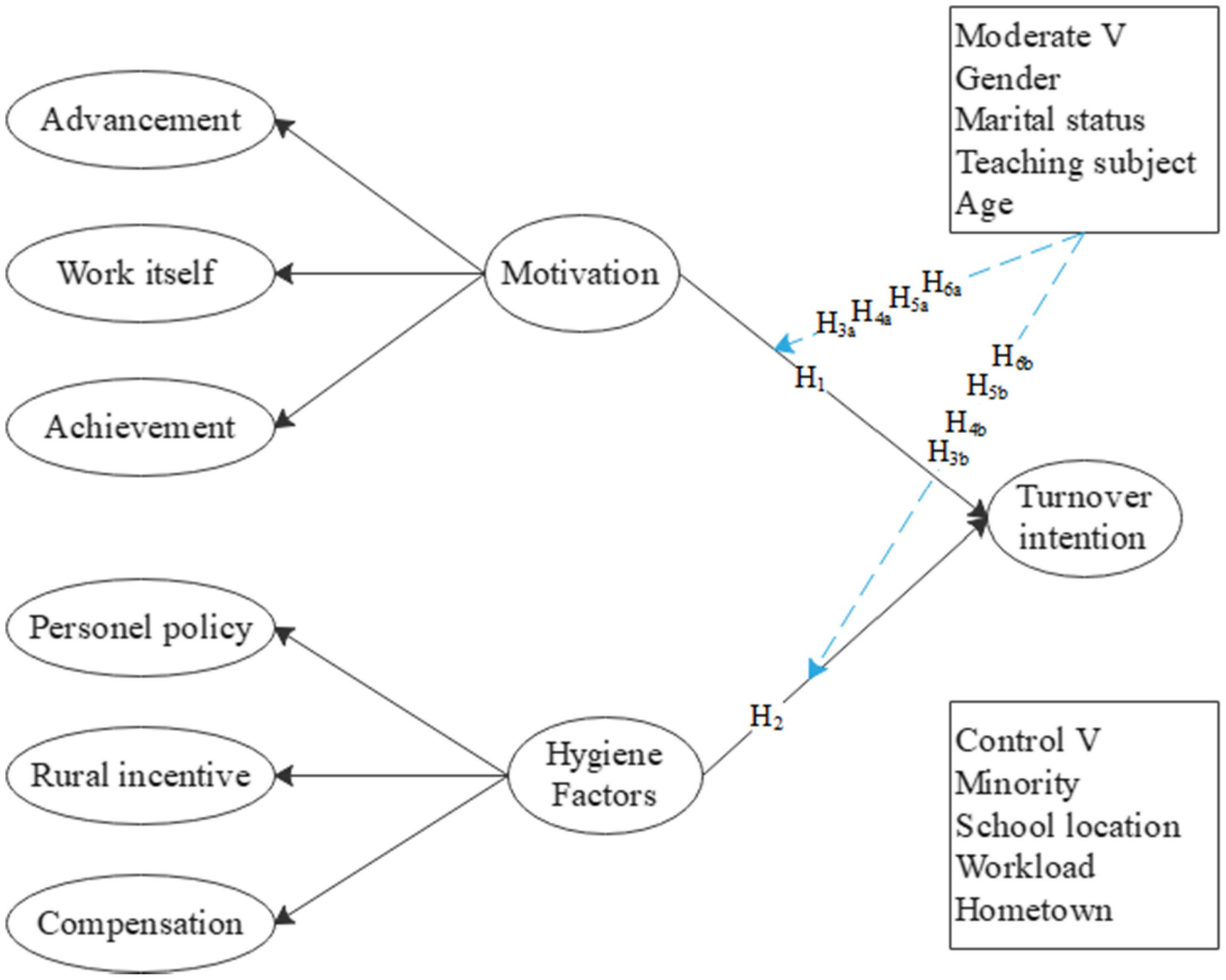
Research model.

Influenced by personal preferences, individual characteristics, etc. there are individual differences in teachers’ work motivation, job satisfaction, and turnover intention. Gender, age, marital status, and teaching subjects may all affect teachers’ turnover intention (Guarino et al., 2006; Shirol, 2014; Geiger and Pivovarova, 2018; Suárez and Wright, 2019). Studies have shown that female teachers have a stronger willingness to move than male teachers at the beginning of their careers, but when teachers are in the middle and late stages of their careers, the turnover tendencies of male teachers and female teachers gradually tend to be the same, and the turnover intention of teachers shows a “U”-shaped trend with the increase of teaching age (Ingersoll, 2002). Scholar Du Ping studied the relationship between the salary of rural primary and secondary school teachers and turnover intention and found that marital status has a significant impact on teachers’ turnover intention, among which male teachers and unmarried teachers have the higher turnover intention (Ping and Yao, 2019). Due to the general shortage of rural art teachers and English teachers, this study focuses on the moderating effects of these two disciplines on the influence of incentive hygiene factors on teachers’ turnover intention. Therefore, the following assumptions are made, see Figure 1:

*H3a*: Gender moderates the strength of the relationship between hygiene factors and turnover intention.

*H3b*: Gender moderates the strength of the relationship between motivation factors and turnover intention.

*H4a*: Marital status moderates the strength of the relationship between hygiene factors and turnover intention.

*H4b*: Marital status moderates the strength of the relationship between motivation factors and turnover intention.

*H5a*: Teaching subjects (art and English) moderates the strength of the relationship between hygiene factors and turnover intention.

*H5b*: Teaching subjects (art and English) moderates the strength of the relationship between motivation factors and turnover intentions.

*H6a*: Age moderates the strength of the relationship between hygiene factors and turnover intention.

*H6b*: Age moderates the strength of the relationship between motivation factors and turnover intention.

## Data sources and research methods

### Sampling method and data collection

The present study was a part of a larger research project on attracting and retaining rural teachers conducted by the first author. The research population targeted in-service teachers working at rural schools in Western China. Multi-step stratified sampling was used for reaching the participants from May to July 2018. First, we choose three areas 14 poverty-stricken areas in Western China based on the poverty area list. Second, we choose one typical county from each poverty-stricken area. Third, we choose rural schools located in nearby counties, remote towns, and the remoter town. At last, we send out 1,000 questionnaires to all teachers in the sampling schools with the help of a local county bureau officer and got 973 valid questionnaires.

In addition, The Research project team also conducted a field visit and in-depth interviews with the relevant persons, such as school principals, teachers, the Bureau Officer of county Education, and students.

### Research objects

Among all valid questionnaires, there were 545 (56%) female teachers, 428 (44%) male teachers, and 788 (81%) minority teachers. The age of teachers is mainly concentrated 34 years old and below and 34 ~ 45 years old. Among them, 315 teachers (32.4%) are 34 years old and below, and 380 teachers are between 34 and 45 years old (39.1%). Married teachers accounted for the majority of 841 (86.4%), and unmarried teachers accounted for 132 (13.6%). In addition, there are 402 (41.3%) teachers in schools around the villages, 324 (33.3%) and 247 (25.4%) teachers in the remoter and remotest villages, respectively. There are 664 (68.2%) teachers whose hometowns are places of residence, and 309 (31.8%) whose hometowns aren’t places of residence, as shown in Table 1.

**Table 1:** Demographic profile of participants (*n* = 973).

Demographics	Category	Frequency	Percentage (%)
Gender	Male	428	44
	Female	545	56
Minority	Yes	788	81
	No	185	19
Age	34 and below	315	32.4
	35–45	380	39.1
	46 and above	278	28.5
Marital status	Married	841	86.4
	Unmarried	132	13.6
School location	Around the villages	402	41.3
	Remoter villages	324	33.3
	Remotest villages	247	25.4
Hometown	Hometowns aren’t places of residence Hometowns are places of residence	309	31.8
		664	68.2

### Variables and measurements

The 18 measurement items in this study were summarized from the existing literature and partially adjusted according to the current research. The projects were based on validated instruments previously (Smerek and Peterson, 2007; Lundberg et al., 2009). All items used a 5-point Likert scale (1 = strongly disagree; 5 = strongly agree). Hygiene factors are divided into three parts: rural incentive policy, personnel policy, and teacher pay. According to policies formulated by most western counties, rural incentive policies are measured according to the rural incentive policies formulated by most western counties (Jiang and Mingze, 2019; Liang, 2021). The three personnel policies are from Anzia and Moe and have been modified according to the actual situation of rural teachers in China (Anzia and Moe, 2013). Finally, the salary evaluation adopts three items of Ryan and adjusts it according to the situation of rural teachers (Ryan et al., 2017).

Motivation factors are divided into three parts: advancement, the work itself, and achievement. All motivation factors are drawn from domestic and foreign literature and adjusted according to the current research context (Sanjeev and Surya, 2016; Qingye, 2018; Zhihui and Weihong, 2018). Teachers leaving their current school or quitting the teaching profession completely represent two aspects of turnover intention (Lindqvist et al., 2014). In addition, moderator variables are selected from individual variables, such as gender (1 = male, 0 = female), marital status (1 = married; 0 = unmarried), art teacher (1 = yes, 0 = no), English teacher (1 = yes, 0 = no), age group (1=under 35 years old,0=over 35 years old). Minority, school location, workload, and hometown were selected as control variables for this study (Zhichun and Jiang, 2007; Perryman and Calvert, 2019).

## Research results

### Reliability and validity test of the measurement model

In this study, higher-order confirmatory factor analysis (CFA) was used to test the reliability and validity of the scale, respectively. The results showed that 16 of the 18 measurement indicators had factor loadings greater than 0.70, and only two indicators had slightly lower factor loading, 0.678, and 0.679, respectively. The t-value shows that each index is significantly correlated with its respective dimensions (*p* < 0.001), which indicates that all the indexes selected in this study have a satisfactory level of reliability. In addition, the overall Cronbach α of the questionnaire is 0.831, only the Cronbach α of the work itself is 0.678, and the rest of the items all exceed the critical value of 0.70, indicating that the internal consistency of each dimension is good. The multiple correlation coefficients (*R*^2^) of all measures ranged from 0.334 to 0.899, indicating that the percentages of these measures that could be explained by predictors ranged from 33.4 to 89.9% (see Table 2). In addition, the Kaiser-Meyer-Olkin value of the questionnaire design in this study was 0.877 > 0.6, and the value of p of the significant probability was 0.000 < 0.01. The sample validity was high, which was analyzed by the validity test.

**Table 2:** The measurement model statistics.

Variables	Items	Loadings	*R* ^2^	*t-*value	Cronbach’s Alpha
**Motivation factors**					
Advancement	I can enlist my colleague’s support in times of professional difficulties.	0.743	0.552	17.897^***^	0.771
	Opportunities for advancement exist in school.	0.749	0.561	17.942^***^	
	I know what is required of me to advance within the school.	0.701	0.491		
Work itself	I enjoy the rural teaching work	0.679	0.461	6.119^***^	0.678
	My teaching job is interesting.	0.756	0.571		
Achievement	The parents of the students I teach support my work	0.708	0.502	13.612^***^	0.762
	My town respects teachers.	0.869	0.755		
**Hygiene factors**					
Rural incentive	The more remote the school, the higher the salary subsidy for teachers.	0.678	0.334	15.052^***^	0.728
	The professional titles of local teachers will be skewed toward rural teachers.	0.713	0.509	17.2^***^	
	Incentives are given to teachers who have been teaching in rural areas for a long time.	0.769	0.591		
Personnel policies	The teacher transfer system of local educational institutions is reasonable.	0.753	0.566	20.694^***^	0.782
	Rural teachers’ promotion policy is fair.	0.72	0.537	20.241^***^	
	The evaluation of teacher performance is reasonable.	0.733	0.518		
Compensation	My salary is competitive.	0.804	0.646	31.992^***^	0.894
	My rural living allowance is appropriate.	0.877	0.769	36.950^***^	
	The benefits package meets my needs.	0.899	0.808		
Turnover intention	I want to leave the teaching position.	0.807	0.651	9.621^***^	0.783
	I want to leave my position at another school.	0.788	0.62		

Significance level of ***p < 0.01.

### The correlation between motivation-hygiene factors and turnover intention

To examine the influence of motivation-hygiene factors on the turnover intention of teachers in western rural areas, a correlation test was carried out. Table 3 shows the correlation between the various dimensions of motivation-hygiene factors and turnover intention. According to the results of the Kendall harmony coefficient, the dimensions of hygiene factors were significantly negatively correlated with turnover intention, and the dimensions of motivation factors were also significantly negatively correlated with turnover intention. And among the hygiene factors, personnel policy has the strongest correlation with turnover intention, *r*_5 = −0.322; among motivation factors, the work itself has the strongest correlation with turnover intention, *r*_2 = −0.412.

**Table 3:** Construct correlations.

Constructs	Mean	Standard deviation	(1)	(2)	(3)	(4)	(5)	(6)	(7)
(1) Advancement	3.338	0.981	1.000						
(2) Work itself	2.290	0.987	0.199	1.000					
(3) Achievement	3.151	1.006	0.561	0.251	1.000				
(4) Rural incentive	2.968	1.073	0.510	0.133	0.445	1.000			
(5) Personnel policy	2.915	1.048	0.694	0.255	0.685	0.602	1.000		
(6) Compensation	2.557	1.039	0.538	0.258	0.520	0.568	0.820	1.000	
(7) Turnover intention	2.781	1.112	−0.289	−0.412	−0.204	−0.177	−0.322	−0.200	1.000

The mean values of each dimension of incentive factors, hygienic factors, and turnover intention are between 2.290–3.338, among which the mean value of advancement is the highest, and the mean value of work itself is the lowest. The standard deviations of incentive factors, hygienic factors, and turnover intention are between 0.981–1.112, among which turnover intention has the highest standard deviation and advancement has the lowest standard deviation.

### Structural model evaluation and correction

According to the research hypothesis, this study constructed a structural equation model of motivation factors, hygiene factors, and teachers’ turnover intention. To evaluate the structural equation model and obtain the path coefficient and its significance level, a preliminary structural model fit evaluation was performed on 973 samples. The test results show that χ^^2^/df = 7.73, RMSEA = 0.08, GFI = 0.91, AGFI = 0.88, and CFI = 0.88. Except for the good fit of RMSEA and GFI, the fit of other indicators is less than 0.9 but still meets the Baumgartner recommendation above 0.8, which may be due to the large sample size (Baumgartner and Homburg, 1996; Jackson et al., 2009). Next, this study will add control variables to the initial model, and use the Bollen-Stine method to correct the fitness of all models (Bollen and Stine, 1992; Fisher and King, 2010), proving that the low fitness of the model is not caused by the poor fitness of the model itself.

To investigate whether there are significant differences in the turnover intention of rural teachers in Western China with different ethnic minorities, school locations, workload, and hometowns. This study conducted a one-way analysis of variance on 973 samples. There are significant differences in teachers’ turnover intention. Therefore, ethnic minorities’ school location workload and hometown were introduced into the structural equation model as control variables. After the introduction of control variables, the model fit index χ^^2^/df = 6.96, which still did not meet the standard requirement of 5.0, but was lower than the χ^^2^/df of the initial model. Second, while GFI = 0.89, AGFI = 0.86, CFI = 0.84, RMSEA = 0.078. Although GFI, AGFI, and CFI all decreased slightly, according to existing research, in addition to motivation and hygiene factors that may have an impact on turnover intention, it is undeniable that the individual characteristics of teachers do have a certain impact on the dependent variable turnover intention.

According to the Bollen-Stine Bootstrap report, the model has a good fitting effect in 2,000 Bootstraps. The poor fit is 0 times, and Bollen-Stine bootstrap = 0.00 < 0.05, which proves that the model is well fitted. Also, the chi-square before correction is 1234.90, while the chi-square after correction is 205.34, which is a big improvement. The modified fit indices are shown in Table 4. It can be found that each fit index has reached the ideal standard value, which is a very obvious improvement compared with fit indexes before the correction. The χ^^2^/df before the modification is 6.82, and it is not only smaller than the ideal standard value of 5.0 but also smaller than the idealized standard value of 3.0. The corrected RMSEA is not only less than the standard of 0.1 but even close to 0. GFI and AGF are less than or equal to 0.9 before the correction, and are not only greater than the standard of 0.9 but also gradually approached 1 after the correction. The CFI is even more equal to 1 after correction. This shows that the model has a very good fit.

**Table 4:** Bollen-Stine bootstrap revised estimates.

Fit indices	Criteria	Before correction	After correction	Fit after correction
χ^^2^ (df)	*p* > 0.05	0.00	0.00	Well fitted
CMIN/DF	<5.0	6.96	1.13	Ideal
RMSEA	<0.1	0.08	0.01	Ideal
GFI	>0.9	0.89	0.97	Ideal
AGFI	>0.9	0.86	0.96	Ideal
CFI	>0.9	0.84	1.00	Ideal

### Path analysis of the structural model

**Figure 2:**
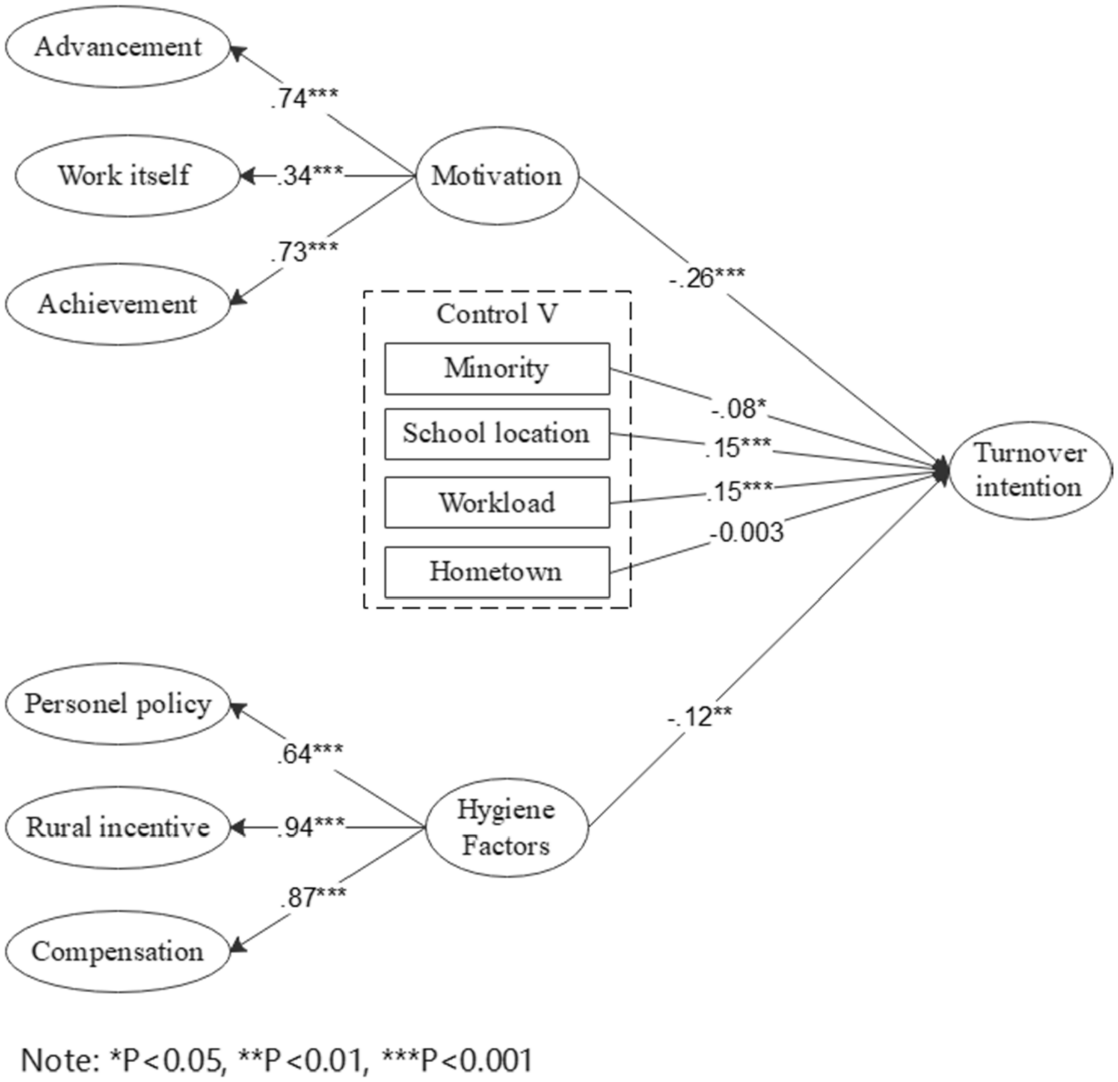
Standardized estimates from structural equation models for teachers’ turnover tendency in Western Rural areas.

The standardized path coefficients among the variables are shown in Figure 2. The motivation factors consisting of promotion, work itself, and achievement and the hygiene factors consisting of rural incentive policies, personnel policies, and remuneration all have significant negative effects on the turnover intention of rural teachers in western China. The standardized coefficients are −0.26 and − 0.12, respectively. Both H1 and H2 are supported by the test results, and the influence of motivation factors on turnover intention is greater than that of hygiene factors, as shown in Table 5. After adding control variables, ethnic minorities, school location, and workload all have significant effects on turnover intention, and their standardized coefficients are −0.08, 0.15, and 0.15, respectively. Among them, hometown has no significant impact on turnover intention. It can be seen that it is difficult to retain rural teachers only by relying on abundant monetary incentives, and non-monetary incentives such as the realization of self-worth, and the satisfaction of emotional needs, can affect rural teachers’ work behavior and decision to leave.

**Table 5:** Structural model path coefficients.

Path	Standardized path coefficient	Unstandardized path coefficient	S.E.	C.R.	*P*	Outcome
Motivation Factors→Turnover Intention	−0.26	−0.32	0.07	−4.86	***	Supported
Hygiene Factors→Turnover Intention	−0.12	−0.12	0.04	−3	**	Supported

Significance level of **p < 0.05, ***p < 0.01.

### The moderating effect of teachers’ marital status and teaching subjects

According to the results of multi-group analysis, it is investigated whether H3-H6 is established, and then the moderating effect of individual factors on the turnover intention of rural teachers in western my country is analyzed. First, this study uses the chi-square test to compare different models to test the path differences between different groups, that is, the strength of the relationship between structures, such as the moderating effect of male and female, married and unmarried, different teaching subjects groups and different ages on turnover intention. The results show that, except for H3a and H3b, 6 of the 8 hypotheses have been verified, and the structural model coefficient of gender = 0.29 > 0.05, indicating that gender does not significantly moderate the relationship between motivation-hygiene factors and turnover intention effect.

The estimation results of the multi-group analysis show that H4a/H4b H5a/H5b and H6a/H6b are supported and the results are significant, as shown in Table 6, and the standardized and unstandardized estimates of the paths between motivation factors, hygiene factors, and turnover intention are given, respectively. The results of the moderating effect show that there are significant differences in the selected moderating variable paths between the variables of the married and unmarried groups, English teachers and non-English teachers, art teachers and non-art teachers, and teachers over 35 years old and teachers under 35 years old.

**Table 6:** Estimates from the multi-group SEM.

	Path	Std. *β*	UNstd β	S.E.	△DF	△CMIN	*P*	△NFI	△TLI
Married teacher	Hygiene→TI	−0.102	−0.100**	0.04	2	9.405	0.009	0.001	0
	Motivate→TI	−0.236	−0.397***	0.09					
Unmarried teacher	Hygiene→TI	−0.31	−0.381***	0.112					
	Motivate→TI	−0.426	−0.822***	0.306					
English teacher	Hygiene→TI	−0.33	−0.470***	0.153	2	5.726	0.057	0.001	0
	Motivate→TI	−0.008	0.004	0.053					
Non-English teacher	Hygiene→TI	−0.105	−0.117**	0.047					
	Motivate→TI	−0.379	−0.761***	0.124					
Art teacher	Hygiene→TI	−0.14	−0.151*	0.089	2	9.881	0.076	0.001	0
	Motivate→TI	−0.562	−1.931***	0.657					
Non-art teacher	Hygiene→TI	−0.132	−0.166***	0.054					
	Motivate→TI	−0.302	−0.618***	0.117					
Under 35 years old	Hygiene→TI	−0.098	−0.124*	0.058	2	3.993	0.036	0.001	0
	Motivate→TI	−0.225	−0.320***	0.089					
Over 35 years old	Hygiene→TI	−0.131	−0.138*	0.067					
	Motivate→TI	−0.457	−0.652***	0.136					

Significance level of *p < 0.1, **p < 0.05, ***p < 0.01.

First, the hygiene factor of unmarried teachers has a significantly stronger influence on turnover intention than married teachers (*β* = −0.31 and − 0.102, *p* < 0.05), and the influence of unmarried teachers’ motivation factors on turnover intention is also significantly higher than that of married teachers. Marriage teachers (*β* = −0.426 and − 0.236, *p* < 0.05). Compared with married teachers, unmarried rural teacher’s pay more attention to institutional environmental factors, and are eager to improve their teaching subjective initiative, attach great importance to personal development, and better devote themselves to rural education construction and development.

Secondly, English teachers paid more attention to hygiene factors than non-English teachers (*β* = −0.33 and − 0.105, *p* < 0.05); art teachers’ motivation factors and hygiene factors had significantly stronger effects on turnover intention than non-art teachers, but art teachers placed more importance on motivating factors (*β* = −0.562 and − 0.302, *p* < 0.01). The results of the study may be related to the higher opportunity cost of English teachers teaching outside the school. At the same time, the characteristics of art education and art teachers should be fully taken into account. The higher investment in the early education cost of art teachers leads art teachers to think that motivation factors are more effective to keep them in their current teaching positions.

Finally, the motivation factors and hygiene factors of teachers over the age of 35 have a stronger impact on turnover intention than those of teachers under the age of 35, and the impact of motivation factors of teachers over the age of 35 on turnover intention is significantly stronger than that of teachers under the age of 35 (*β* = −0.457 and − 0.225, *p* < 0.05). Likewise, the effect of hygiene factors on turnover intention was stronger for teachers over 35 years old than for teachers 35 and under (*β* = −0.131 and − 0.098, *p* < 0.05). Teachers of different age groups pay more attention to the satisfaction of motivating factors. If rural schools pay more attention to the motivating factors that teacher’s value, rural teachers are more willing to stay and continue teaching.

## Conclusion and discussion

### Conclusion

Drawing on motivation-hygiene theory, this study theoretically and empirically tests a model of hygiene and Motivation factor that explains teachers’ turnover intention and examines the moderation effect of gender, marital status, and teaching subject, respectively. Three major findings from this study are as follows:

First, Hygiene factors, composed of compensation, incentives, and personnel policy are the significant predictor which restrains teachers’ turnover intention. Particularly, personnel policy is the most influential factor in building hygiene factor. This finding implies that rural teachers in China indeed respond to the three key hygiene factors, and their turnover intention can be decreased by raising compensation level (Shen, 1997; Stinebrickner, 1998; Podgursky et al., 2004), attractive rural incentives (McEwan, 1999) and reasonable personnel policy (Loeb et al., 2009; Anzia and Moe, 2013).

Second, Motivator factors composed of achievement, work itself, and advancement, are negatively related to rural teachers’ turnover intention. Besides, the impact of motivational factors on turnover intention is greater than that of hygiene factors. Among the three kinds of Motivator factors, Advancement contributes the most as a factor. Thus, rural schools providing internal growth opportunities such as training, and teaching instruction, would increase teachers’ teaching skills and competence, which results in lower teacher turnover (McGilton et al., 2013). In addition, schools providing teacher respect, recognition, and appreciation may also reduce rural teachers’ turnover intention (Hogan et al., 2013).

Third, marital status, teaching subject, and age have a significant moderation effect on the relationship between motivation/hygiene factors and turnover intention, whereas gender has no significant moderation effect. Both the hygiene and motivator factor are significantly stronger among unmarried teachers than married teachers. English teachers concern more on hygiene factors, which affect their turnover intention stronger than non-English teachers. For art teachers, both the effect of hygiene and motivation factors on turnover intention is significantly stronger than for non-art teachers. The motivation factors and hygiene factors of teachers over 35 years old have a stronger impact on turnover intention than those of teachers under 35 years old.

### Theoretical implications

First, this study enriches teacher turnover intention literature by exploring the effect of institutional factors in the context of the Chinese western rural labor market. Previous studies of teachers’ turnover intention are based on a set of factors including personal attributes, school conditions, and family support, respectively, (Yanling et al., 2017). However, institutional factors especially rural teacher incentives have not been thoroughly explored. Our study sorted the institutional factors from a theory perspective and examined their influences on rural teachers’ turnover in western China.

Second, by adopting the hygiene-motivation theory, this research empirically verifies three types of hygiene factors and motivator factors with distinct effects on turnover intention. The previous study mainly tested this theory on employees of the private sector, while only a few directly examine the campus teacher. Our research identifies different weighted effects of Hygiene and Motivator factors on turnover intention and extends the current turnover intention literature to the context of the rural teacher labor market.

Third, this study extends the Hygiene-motivator theory by examining the moderation of personal factors between teacher motivation and turnover intention in the Chinese context. A limited study is concerned with the moderation effect of individual factors. Our research verifies the effect of hygiene and motivator factors on turnover intention differed between married teachers and unmarried teachers, which supports Ping and Yao (2019) conclusion. Besides, a distinct effect also exists between teachers of different ages, English teachers, non-English teachers, art teachers, and non-art teachers in rural China.

### Implications for attracting and retaining rural teacher

Our findings may have important implications for relevant education practitioners to better manage the rural teachers’ turnover problems in less developed countries.

First, policymakers should emphasize the influence of hygiene factors on teachers’ turnover decisions, especially design a reasonable and flexible personnel policy for rural teachers. For example, the performance evaluation plan should aim to help rural teachers to improve their teaching skills and ability not just assess them. Also, the county government should make a more flexible teacher exchange plan to help teachers to transfer their job between rural schools and urban schools. Besides, our results have verified that the various rural incentive policy enacted in Chinese has the effect to reduce teachers’ turnover intention. Hence, the county government should continue to strengthen the monetary and non-monetary incentives for rural teachers, such as increasing the standard of subsidy for rural, more chances for rural teachers to have promotions, and so on.

Meanwhile, it is worthwhile noting that motivator factors play a more important role in restraining teachers’ turnover intention, especially the advancement factor. This finding implies rural schools should focus on rural teachers’ internal growth opportunities, such as training, and teaching instruction, to increase teachers’ teaching skills and competence. Besides, recognizing employees’ effort and contribution is also an effective and less expensive approach to attracting rural teachers for school managers.

At last, both the hygiene and motivator factor are significantly stronger among unmarried teachers than married teachers. This result implies that policymakers should pay more attention to unmarried teachers’ hygiene and motivation needs, including career development, recognition, and incentive. Besides, English/art teacher’s pay more attention to hygiene factors than non-English/art teachers, this may be because the salaries of English teachers in rural areas are relatively insufficient compared with civil servants (Soodmand Afshar and Doosti, 2016). If English /art teachers choose to work in the off-school tutoring institution, they can get more compensation. Therefore, the opportunity cost for English teachers to teach in other schools will increase accordingly as well as for art teachers. So policymakers should design art and English teacher’s salary above the market level. Finally, the results of age group adjustment show that, for rural teachers of different age groups, improving hygiene factors and motivating factors can significantly reduce their turnover intention, and the effect of motivation factors is much greater than hygienic factors. In addition, the same motivation and hygienic factors have a greater effect on teachers over the age of 35. It can be seen that the various reform policies implemented for rural teachers have a better retention effect on teachers over the age of 35.

### Limitations

This study contains limitations that can turn into suggestions and opportunities for further research. First, the present study only examined the individual perception of hygiene and motivator factors and turnover intention. These variables can also be estimated from a school perspective, so future research can apply a school-level analysis to investigate the effect of hygiene and motivation factors on a school’s turnover rate. Second, despite our best effort to incorporate the three kinds of hygiene-motivator factors into the model according to our investigation, we cannot exclude the possibility of having other important factors that may also contribute to the hygiene-motivator construct. For example, our model did not consider the effect of the working condition of the school, which also play a key role in turnover intention (Boyd et al., 2009; Bullough et al., 2014). Third, this study is not comprehensive as some mediating variables were not included in the research model, for example, job satisfaction, and self-efficacy. At last, the generalizability of our results is limited. As we only collected data from western China, the sample may not be representative enough of eastern and middle China.

## Data availability statement

The datasets presented in this article are not readily available because of privacy concerns to local legislation. Requests to access the datasets should be directed to yukairuikerry@163.com.

## Author contributions

JJQ conceived the original idea. JJQ and YJ analyzed the data. YJ, YKR, and LCN wrote the original manuscript with input from all authors. JJQ, YKR, and YJ contributed to the literature review. YKR and LCN reviewed and edited the final manuscript. JJQ supervised the study. All authors discussed the results and contributed to the final manuscript.

## Funding

This research project is funded by the National Social Science Fund Youth Project of China “The construction and application of China’s teacher status index” (project number: CFA210244).

## Conflict of interest

The authors declare that the research was conducted in the absence of any commercial or financial relationships that could be construed as a potential conflict of interest.

## Publisher’s note

All claims expressed in this article are solely those of the authors and do not necessarily represent those of their affiliated organizations, or those of the publisher, the editors and the reviewers. Any product that may be evaluated in this article, or claim that may be made by its manufacturer, is not guaranteed or endorsed by the publisher.
